# Ready to screen? Start with the goal in mind

**Published:** 2017-08-07

**Authors:** Clare Gilbert

**Affiliations:** 1 MB ChB, FRCOphth, MSc (Epid), MD – Co-Director Disability and Eyes (ICEH) Group.

**School eye health programmes must be planned properly. Before any work begins, everyone needs to be clear on what the objectives are and how the team will monitor their progress. Only then will programmes achieve their maximum impact.**

**Figure:**
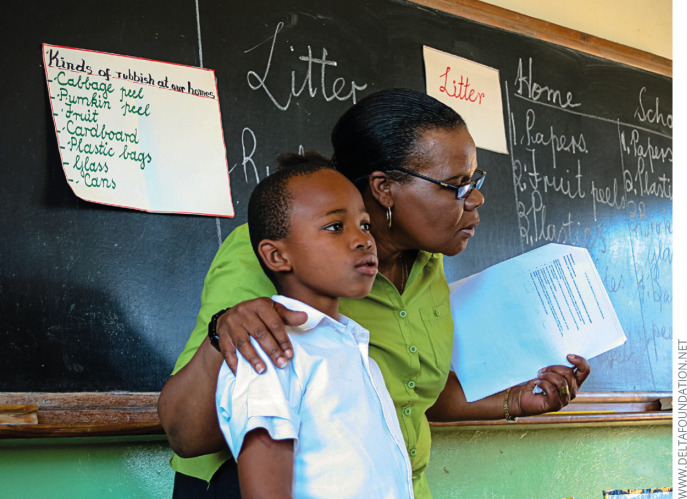
School teachers may need eye care too.

As with any planning, it is important to decide what is to be achieved by school eye health initiatives. Before acting, it is important to ask the question “What is the benefit we hope to achieve, and for whom?” This positive change is the goal or impact of the programme. It is important to ask this question early on when planning school eye health programmes. Depending on the goal, several different activities are possible, each of which would benefit different groups of people.

## Activities to benefit school children

- Detect and treat refractive errors in school children
- Detect and refer children identified with other eye conditions
- Train school nurses to detect and treat simple eye conditions in children in schools
- Health education on how children can keep their eyes healthy

## Activities to benefit teachers

- Detecting and treating refractive errors, including presbyopia, in teachers
- Detecting and referring teachers who have poor vision or a history of cataract, glaucoma or diabetic retinopathy.

## Activities to benefit communities

- Teaching children how to be agents of change for healthy eyes in their families and communities (see **www.childtochild.org.uk**).

Having decided which components to include, the activities needed to achieve the goal can then be planned, working **backwards** from the goal/impact. It is important to describe the objectives, which, if implemented, would bring about the goal/impact intended. The objectives need to be clear, precise and measurable. The term SMART is often used: Specific, Measurable, Achievable, Relevant and Time-bound.

## Example

Imagine a school eye health programme with the following goal: “Less visual impairment from uncorrected refractive errors and other eye conditions in children, better near visual functioning among teachers and a lower risk of visual loss from diabetic retinopathy among teachers with diabetes.”

**Figure 1:**
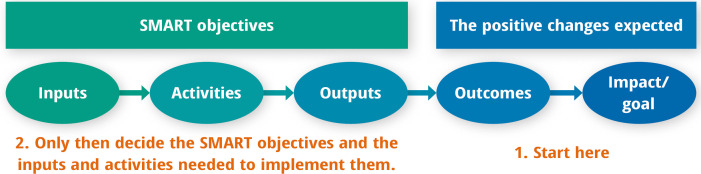
Overview of planning school eye health programmes

**Figure 2:**
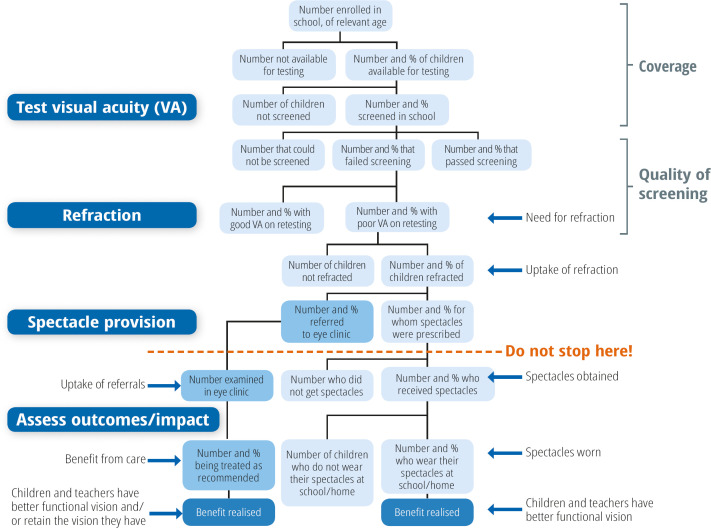
Data to be collected (for children and teachers) to monitor and evaluate a school eye health programme at each stage.

To achieve this goal, the objectives might be as follows.

1. To train {xx} teachers in {xx} schools to screen visual acuity in children
2. To screen {xx} children aged 10–18 years between {date} and {date}
3. To dispense spectacles for distance correction to {xx} (y%) children, {xx} (y%) pairs of which will be ready-made spectacles
4. To refer {xx} (y%) children to the base hospital for assessment and treatment
5. To refer {xx} teachers (y% of those aged 40 and above who have diabetes) for diabetic retinopathy screening, and treatment if required

The numbers (xx's) will vary from place to place and should be based on information from publications and/or reports of previous school eye health programmes in the same or a similar location.

The activities to implement the objectives are shown in the panel (right). Once the activities have been planned, the inputs needed to deliver them can be identified and budgeted for. The objectives are also important as they generate the indicators needed to monitor progress in implementing the programme (see panel, p. 27).

**Activities based on objectives 1–5, and monitoring data to be collected**

In this example, the activities include the following.

- Training teachers in screening vision at the 6/9 or 6/12 level in each eye
- Vision screening and simple eye examination of children
- Recording the visual acuity measurement, refraction and best corrected visual acuity of those who fail screening
- Prescribing and dispensing spectacles (custom-made or ready-made) to children who fulfil the prescribing guidelines
- Referring children whose vision does not improve with refraction, who have eye abnormalities, or who need cycloplegic refraction
- Assessing near acuity in teachers using standard methods and dispensing near reading spectacles if needed
- Counselling and referring all teachers known to have diabetes for a retinal examination.

The objectives guide the data that need to be collected (the indicators) to continuously monitor progress and assess the quality of the programme. Examples are:

- The number of teachers trained
- The number of children screened
- The number of children prescribed spectacles
- The number of children referred.

**Figure:**
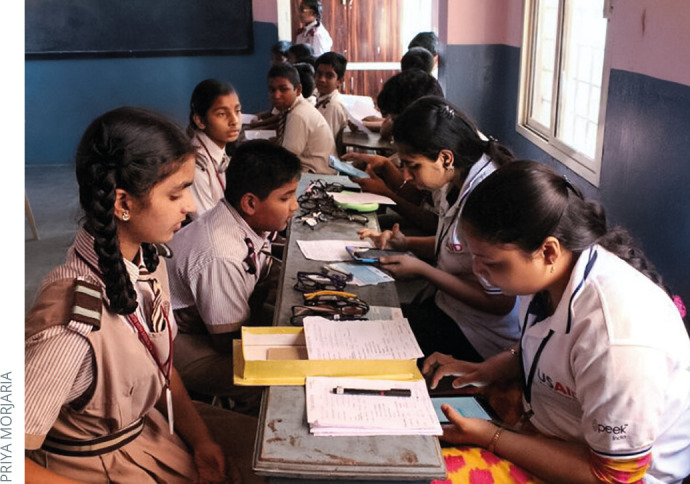
Delivering spectacles to children in schools is recommended. INDIA

Most school eye health programmes do not collect any data beyond the number of pairs of spectacles dispensed/given. This means that it is not possible to assess whether the programme has had any impact, i.e. whether it has made any difference to the lives of children or teachers (see the line “Do not stop here!” in Figure 2, p. 27).

Without knowing the number and proportion of teachers referred who attend the base hospital and then undergo treatment, we have no idea whether they have benefited - we assume they do. Similarly, without recording the number of children who obtain spectacles and the number and proportion who subsequently wear them, we cannot assess the impact of the programme.

We often make assumptions that children will obtain and then wear their spectacles when, in fact, they may not do so in large enough numbers to make the activity worthwhile. We also often make assumptions that children and teachers function better and are satisfied with the treatment they have received as a result of the programme. However, we cannot know this without gathering the appropriate evidence.

**Table 1:** Using data (see Figure 2) to identify problems. Only 28 of the estimated 400 children in this example benefited from improved vision

**Actual need**		
Total number of children	10,000	
An estimated 4% have significant RE	**400**	**Need spectacles**
**Monitoring data**		
Total number of children	10,000	
80% (of 10,000) screened	8,000	
8% (of 8,000) failed screening	640	
60% (of 640) attended for refraction	384	
60% (of 384) were prescribed spectacles	230	
40% (of 384) had normal vision on retesting	154	*[False positives]*
**Outcome data**		
50% (of 230) received their spectacles	115	
30% (of 115) wear their spectacles	**35**	**Wear spectacles**
**Impact data**		
80% (of 35) report better vision	**28**	**Satisfied**

Additional information can readily be collected to assess the coverage of the programme and the quality of screening (Figure 2, p 27).

## How to use information collected during school eye health programmes

So why do we need to do all this? The answer is that measuring what we are doing allows us to see how we can improve. For example, if none of the teachers identified as having diabetes attend the base hospital for retinal examination, we need to know this. We must either find out why this has happened so that corrective action can be taken, or stop doing it. If the programme includes children aged 5–9 years, it is important to know what proportion of them need and subsequently wear their spectacles. If this is very low then a decision needs to be made whether including this age group is a good use of resources.

Suppose we plan to screen 10,000 children aged 10–15 years, and we estimate that 4% have significant uncorrected errors (Table 1). This means that there are around 400 children needing spectacles in this population of children. As a result of careful monitoring we find out that 4 schools did not participate, and only 8,000 children were screened. 8% of the 8,000 children screened failed screening, but only 60% of these attended for refraction. Among the children refracted, some had normal vision on retesting and the others were dispensed spectacles. Among the latter, only 50% obtained their spectacles, and only 30% of the children who obtained spectacles were actually wearing them 4 months later. Not all (80%) were satisfied with their spectacles. As can be seen from Table 1, this means that although the intention had been that 400 children would benefit, only 35 were wearing their spectacles, and only 28 were satisfied with them: this is only 7% of the 400 we anticipated would benefit. A similar set of information should be collected for children referred with other eye conditions to eye care providers. If uptake is low, then the reasons need to be explored so that corrective action can be taken.

The data in Table 1 raise a lot of questions which, if addressed, could improve the programme. Improving coverage may require better explanation of the programme to headteachers, or flexibility as to when the programme might take place. In this example, approximately twice the proportion of children (8%) failed screening than anticipated. A high proportion of those who failed screening and came for refraction (40%) did not need spectacles. This suggests that vision screening could be improved (see article on p. 31), and where and/or when refraction is done may need to be rethought. Only half of the children prescribed spectacles actually obtained them, which is a common finding. Ways to improve immediate access to affordable, high quality spectacles need to be explored (see article on p. 33).

In summary, school eye health programmes need to be carefully planned and monitored so that the maximum benefit can be achieved for the resources available.

